# Statins and prostate cancer—hype or hope? The epidemiological perspective

**DOI:** 10.1038/s41391-022-00554-1

**Published:** 2022-06-22

**Authors:** Emma L. Craig, Konrad H. Stopsack, Emma Evergren, Linda Z. Penn, Stephen J. Freedland, Robert J. Hamilton, Emma H. Allott

**Affiliations:** 1 Patrick G Johnston Centre for Cancer Research, Queen’s University Belfast, Northern Ireland, UK; 2 Department of Epidemiology, Harvard T.H. Chan School of Public Health, Harvard University, Boston, MA USA; 3 Princess Margaret Cancer Centre, University Health Network, Toronto, ON Canada; 4 Department of Medical Biophysics, University of Toronto, Toronto, ON Canada; 5 Division of Urology, Department of Surgery, Samuel Oschin Comprehensive Cancer Institute, Cedars-Sinai Medical Center, Los Angeles, CA USA; 6 Section of Urology, Durham Veterans Affairs Medical Center, Durham, NC USA; 7 Department of Histopathology and Morbid Anatomy, Trinity Translational Medicine Institute, Trinity College Dublin, Dublin, Ireland

**Keywords:** Cancer epidemiology, Prostate cancer

## Abstract

**Background:**

Men using cholesterol-lowering statin medications have been found to have lower risks of both advanced and fatal prostate cancer in multiple registry-based studies and prospective cohort studies. Statin use has also been associated with longer survival among men already diagnosed with prostate cancer. Mechanisms responsible for purported anti-cancer effects of statins are not well understood but may offer insight into prostate cancer biology.

**Methods:**

We summarise epidemiological data from studies of statins and prostate cancer and discuss to what extent these findings can be interpreted as causal. Additionally, lipid-mediated and non-lipid-mediated mechanisms that may contribute to potential anti-cancer effects of statins are reviewed. Finally, we consider treatment settings and molecular subgroups of men who might benefit more than others from statin use in terms of prostate cancer-specific outcomes.

**Results:**

Data from prospective observational studies generally reported a lower risk of fatal prostate cancer among statin users. There is some evidence for serum cholesterol-lowering as an indirect mechanism linking statins with advanced and fatal prostate cancer. Window-of-opportunity clinical trials show measurable levels of statins in prostate tissue highlighting potential for direct effects, whilst observational data suggest possible statin-driven modulation of prostate microenvironment inflammation. Additionally, emerging data from registry studies support a potential role for statins within the context of androgen deprivation therapy and anti-androgen treatment.

**Conclusion:**

Prospective and registry-based studies support a lower risk of advanced and fatal prostate cancer in statin users relative to non-users, as well as better outcomes among prostate cancer patients. The few randomised-controlled trials conducted so far have short follow-up, lack identified molecular subgroups, and do not provide additional support for the observational results. Consequently, additional evidence is required to determine which men may experience greatest benefit in terms of prostate cancer-specific outcomes and how statin effects may vary according to molecular tumour characteristics.

## Introduction

Statins are competitive antagonists of 3-hydroxy-3-methylglutaryl coenzyme A (HMG-CoA) reductase, whose activity is rate-limiting for the biosynthesis of cholesterol and derivatives via the mevalonate pathway. While the evidence supporting the role for statins in cardiovascular disease prevention is unequivocal [1], mounting data point to a potential role for statins in cancer prevention, with some of the more promising data in prostate cancer.

This review discusses epidemiological evidence supporting a potential role for statins in prostate cancer prevention, particularly prevention of fatal disease. Whilst underlying mechanisms behind statin effects in cancer are currently unclear, we review evidence from epidemiological studies that may support existing theories and provide greater understanding of prostate cancer biology. This is followed by an evaluation of data exploring the timing of statin use alongside, and potential synergy with, androgen deprivation therapies (ADT). Finally, we review current data regarding which patients may benefit more from statin use. Our companion review discusses mechanistic evidence of anti-tumour effects of statins from laboratory studies [2].

### Statins and fatal prostate cancer

Following the first report of a stronger association of pre-diagnosis statin use with advanced, metastatic and fatal prostate cancer [3], the majority of studies have performed analyses stratified by tumour stage and grade. A meta-analysis of 15 cohort and 12 case-control studies found statin use was associated with 20% (relative risk (RR) 0.80; 95% CI 0.70–0.90) lower risk of advanced prostate cancer [4]. In contrast, associations between statin use and low-risk prostate cancer are generally null [5]. The most pressing question is whether statins could prevent fatal prostate cancer, responsible for 375,304 deaths worldwide (or 31,638 for US) per year [6, 7].

Two prospective cohort studies addressed this question by following cancer-free men long term. Findings from the Health Professionals Follow-up Study, with 801 fatal prostate cancers diagnosed amongst 44,126 men during 24 years of follow-up, showed a 24% lower rate of fatal prostate cancer in pre-diagnosis statin users vs. non-users (hazard ratio (HR) 0.76; 95% CI 0.60–0.96) [8]. In the Atherosclerosis Risk in Communities (ARIC) study, Mondul et al. took a similar approach, this time following men until death, including 90 fatal cancers diagnosed among 6518 men during 20 years of follow-up. Results for the association between statin use and rates of fatal prostate cancer (HR 0.65, 95% CI 0.40 to 1.05) were compatible with the Health Professionals Follow-up Study estimate [9]. These findings from large prospective studies with substantial follow-up support a potential role for statin use in reducing fatal prostate cancer risk, though the optimal timing of exposure remains unclear.

In addition, registry-based studies have explored effects of pre- and post-diagnosis statin use on prostate cancer-specific mortality. A UK registry study reported that the association between post-diagnosis statin use and prostate cancer-specific mortality was strongest amongst pre-diagnosis statin users (HR 0.55; 95% CI 0.41–0.74 in men who used statins pre-diagnosis vs. HR 0.82; 95% CI 0.71–0.96 in men who did not) [10]. In contrast, analyses of the Finnish Randomised Study of Screening for Prostate Cancer and the Danish registry, both described below, reported similar associations between post-diagnosis statin use and prostate cancer-specific mortality irrespective of pre-diagnosis use [11, 12].

Given that any clinical trial of statins is more feasible among men already diagnosed with prostate cancer, it is informative to evaluate the effect of post-diagnosis statin use on prostate cancer-specific outcomes. Indeed, all three aforementioned studies reported similar magnitudes of association between post-diagnosis statin use and reduced prostate cancer-specific mortality. Specifically, the Finnish Randomised Study of Screening for Prostate Cancer showed that post-diagnosis statin use was associated with a 20% (95% CI 0.65–0.98) lower rate of prostate cancer-specific mortality, with stronger associations among those using higher doses of statins and for longer durations [11]. The magnitude of this association was in line with results from the much larger Danish registry study (HR 0.83; 95% CI 0.77–0.89) [12] and the UK registry study (HR 0.76; 95% CI 0.66–0.88) [10]. As such, while statin use prior to diagnosis appears to be associated with better prostate cancer outcomes, post-diagnosis statin use without pre-diagnosis use is associated with ~20% lower rate of prostate cancer-specific mortality.

## Bias in existing studies: do associations have causal interpretations?

Before taking next steps, a central question is whether bias from confounding and other sources may affect reported associations between statin use and prostate cancer outcomes as they do in studies of other commonly used medications [13]. For example, confounding is an intrinsic property of observational analyses where treatment is not randomly assigned. In such cases, statin users and non-users may differ in many factors associated with prostate cancer risk and outcomes that can bias results. Immortal time also notoriously affects many studies, resulting from analytical approaches predicting the past (e.g. surviving the first three years of follow-up) using information from the future (becoming a statin user in year four), making statins appear beneficial [14, 15]. Additionally, most observational studies have followed an etiology-focused paradigm aimed at understating biological effects of statins on prostate cancer outcomes rather than an action-focused paradigm aimed at estimating treatment effects. One exception is an emulated target trial of statin initiation that had null results for total prostate cancer (HR 1.02, 95% CI 0.95–1.09) over 4 years of mean follow-up and no data on advanced or fatal prostate cancer [16].

Appropriate analytic methods as well as prospective study designs help reduce potential sources of bias. Prospective cohort studies address confounding through adjustments for detailed measures of modifiable and non-modifiable risk factors for prostate cancer and competing causes of death, with registry studies using medical diagnoses and medications as proxies. As it is ultimately unknowable if confounding and bias was successfully controlled in observational studies, results from existing randomised trials need to be considered.

The Cholesterol Treatment Trialists’ Collaboration pooled data from 27 major randomised-controlled trials of statin therapy, including 1877 incident prostate cancers and 211 prostate cancer deaths over a median follow-up of 4.8 years, with null results (hazard ratio per 1 mmol/L of LDL cholesterol reduction with statin therapy 0.97, 95% CI 0.85 to 1.10) [1, 17]. A double-blind, randomised-controlled trial of moderate-intensity atorvastatin among 364 men who underwent radical prostatectomy reported a null association with PSA levels over time, with the primary endpoint biochemical recurrence at 1 year (hazard ratio 1.00, 95% CI 0.71–1.41), and following up to 5 years follow-up [18]. With the relatively short follow-up in studies, at this point, randomised-controlled trials lend no further support for the hypothesis that statins may affect prostate cancer mortality, however, the highest-quality observational studies are consistent with an inverse association of statin use with advanced and fatal prostate cancer.

## Observational data compatible with anti-cancer statin mechanisms

Identification of mechanisms linking statins and prostate cancer could provide a biological rationale to support epidemiological associations. Unfortunately, the mechanisms responsible for purported statin anti-cancer effects are still unclear though two broad categories have been proposed: lipid-mediated and non-lipid-mediated, for which we have summarised the epidemiological evidence below. Our companion review considers mechanistic evidence from laboratory studies [2].

## Lipid-mediated statin mechanisms

Statins have a pronounced effect on serum lipid levels, reducing low density lipoprotein (LDL) cholesterol by 30–60%, total cholesterol by 23–28% [19], and triglycerides by 25–45% [20, 21]. Lipid accumulation and deregulated lipid signalling is well-recognised as a hallmark of prostate cancer, so it follows that statins could affect prostate cancer risk indirectly, via their effect on serum and intratumoral cholesterol. This theory is in line with some, though not all, epidemiological studies which report an association between serum lipids and prostate cancer risk, as summarised below.

In a meta-analysis of six studies, the association between high versus low categories of total serum cholesterol and high-grade or advanced prostate cancer (RR 1.32; 95% CI 0.93–1.87) tended to be slightly more pronounced than for total prostate cancer (RR 1.05; 95% CI 0.97–1.14) [22]. Several other large studies were published since, including a case-cohort analysis in EPIC-Heidelberg which found no association between total cholesterol or any cholesterol sub-fraction and total prostate cancer risk [23, 24]. Similarly, a Mendelian Randomisation analysis of genetically-predicted lipid levels in the PRACTICAL consortium reported no significant association between cholesterol and total prostate cancer risk, though they observed weak positive associations for LDL and triglyceride levels with high-grade prostate cancer [23]. In contrast, two additional prospective studies (one not meeting meta-analysis inclusion criteria and one published since), both found positive associations between serum cholesterol and aggressive prostate cancer risk [24]. An analysis of 698 men with prostate cancer and 698 matched controls with measured serum cholesterol in the Health Professionals Follow-up Study showed that men with low total cholesterol were at lower risk of high-grade prostate cancer (odds ratio [OR] 0.61; 95% CI 0.39–0.98) [24], and a secondary analysis of the Reduction by Dutasteride of Prostate Cancer Events (REDUCE) trial reported positive associations between high total serum cholesterol (OR per 10 mg/dl increase 1.05; 95% CI 1.00–1.09) and high-grade prostate cancer [25]. In sum, while the evidence does not support an association of serum cholesterol or cholesterol sub fractions with total prostate cancer risk, there is some support for an increased risk of aggressive disease in men with dysregulated serum cholesterol levels. Some, though not all [26], studies have found an association between higher serum cholesterol levels and increased prostate cancer-specific mortality [27, 28]. Pending further study, modification of serum lipids could be a contributing mechanism to the observed effect of statins in prostate cancer.

An additional consideration is that serum lipid levels may not reflect tumour lipid levels. In a randomised-controlled trial, 160 men were treated with radical prostatectomy after randomisation to high-intensity atorvastatin (80 mg daily) or placebo for at least 28 days [29]. Metabolomics of matched serum and tumour-adjacent normal prostate tissue revealed a shift in both serum and prostate tissue lipidome of those randomised to high-dose atorvastatin. The authors speculated that, of the many metabolites assessed, lower levels of unsaturated lysophosphatidycholines (20:4) and (18:2) in prostate tissue of statin users could hamper tumour cell adaptation to hypoxia. However, which of the lipid species affected by statin use, both in serum and prostate tissue, are most relevant for fatal prostate cancer risk is not yet known.

One approach to teasing apart the mechanism is to compare associations of statin versus non-statin cholesterol-lowering drugs with prostate cancer, though this can be challenging due to the overlap in use of statin and non-statin cholesterol-lowering medications and therefore necessitates large studies. A registry study from the Canadian province of Saskatchewan [30] found that both statin users (HR 0.51; 95% CI 0.41–0.63) and users of non-statin cholesterol-lowering drugs (HR 0.66; 95% CI 0.51–0.85) had lower rates of prostate cancer-specific mortality. A sensitivity analysis of non-statin cholesterol-lowering drug users who did not use statins (42% of non-statin cholesterol-lowering drug users) produced similar results. The authors proposed that the similarity in these estimates supports cholesterol-lowering as the predominant mechanism contributing to the inverse relationship between statins and fatal prostate cancer. However, another study of the Saskatchewan population, albeit using a different sampling and analysis strategy [31], found an association between statin use and decreased risk of clinically significant prostate cancer (OR 0.84; 95% CI 0.73–0.97) which was not observed for non-statin cholesterol-lowering drugs (OR 1.06; 95% CI 0.78–1.45). Additionally, a study by Murtola et al. found no association between pre-diagnosis or post-diagnosis non-statin cholesterol-lowering drug use on prostate cancer-specific mortality, though there was large overlap of these drug categories with 87% of non-statin cholesterol-lowering drug users also using statins [11]. As such, there is some evidence for both lipid and non-lipid-mediated mechanisms contributing to inverse associations between statin use and prostate cancer mortality.

## Non-lipid-mediated statin mechanisms

Several studies have found measurable statin levels within the prostate tissue itself. The ability of statins to access the prostate supports the potential for direct physiological effects of statins unrelated to systemic lowering of circulating cholesterol and other lipid species. Two window-of-opportunity trials measured statin concentrations in prostate tissue after roughly 4 weeks of statin treatment. A Canadian pilot trial randomised men with localised prostate cancer to 80 mg fluvastatin for 4–12 weeks prior to their scheduled radical prostatectomy. Fluvastatin was detected in prostate tissue of 10 (36%) of 28 patients evaluated, and mean intraprostatic fluvastatin concentration in these ten patients was 9.7 ng/g or 24 nM, while mean serum fluvastatin levels were tenfold higher (200 nM) [32]. Another trial in a Finnish population randomised men to 80 mg atorvastatin for a median of 27 days before radical prostatectomy [33]. Atorvastatin was detectable in the prostate of 28 patients (50%), with a median intraprostatic concentration of 17.6 ng/g or 32 nM among those with detectable levels, five times higher than plasma levels (3.6 ng/mL or 6.4 nM). In this study, men with measurable levels of atorvastatin in their prostate tissue used statins for longer and more regularly than those without measurable levels suggesting longer, more regular use of statins may be required for statins to access prostate tissue and thus have direct effects. Alternatively, the ability to measure statins in some patient tissues and not others could be related to mechanisms of statin uptake by tumour cells or time of sampling relative to last dose of statin administration.

### Inflammation

Reduction of systemic and local inflammation is one of the best described non-cholesterol-lowering effect of statin use. Results from secondary analyses of cardiovascular disease clinical trials demonstrate that statins lower plasma levels of the inflammatory biomarker C-reactive protein [34, 35] in a largely LDL-independent manner. Statins have also been shown to reduce local inflammation within the vascular system [36], and in inflammatory diseases such as rheumatoid arthritis [37]. With regards to prostate tissue itself, one of the first observations of a relationship between statin use and prostate inflammation came from a study of men undergoing radical prostatectomy with histological inflammation graded by a pathologist [38]. Statin use relative to non-use in the year before radical prostatectomy was associated with lower risk of histological inflammation surrounding malignant glands in the resected prostate (OR 0.31; 95% CI 0.10–0.98) and this observation was more pronounced among men taking higher doses of statins.

Two prostate cancer chemoprevention trials, REDUCE and the Prostate Cancer Prevention Trial (PCPT), provided opportunities for assessing the association of statin use with benign prostate inflammation among men undergoing study-mandated, PSA-independent prostate biopsies. REDUCE, which recruited men with elevated baseline PSA but a negative prostate biopsy, found lower risk of chronic histological inflammation of negative biopsies in statin users versus non-users (OR 0.81; 95% CI 0.69–0.95) and suggested lower odds of severe acute histological inflammation (OR 0.73; 95% CI 0.53–1.00) [39]. By contrast, PCPT recruited men without indication for biopsy (PSA < 3 ng/ml at baseline) and therefore may be more generalisable to the population, with the caveat that benign regions of prostate tissue were sampled from prostate biopsies positive for cancer. An analysis of the placebo arm of the PCPT, which assessed benign prostate tissue inflammation using histological and immunohistochemistry approaches, reported that while there was no association between statin use and histological inflammation, statin users had lower expression of the macrophage marker CD68 [40].

A window-of-opportunity clinical trial which administered atorvastatin to 158 men scheduled to undergo radical prostatectomy in Finland examined intraprostatic inflammation as a secondary endpoint. Among men with high-grade disease randomised to atorvastatin, histological inflammation score was slightly lower [41]. Finally, an exploratory gene set enrichment analysis of 10 statin users and 103 non-users with prostate cancer reported that T-cell receptor activation was the top differentially expressed pathway associated with statin use, with many of the other significantly altered pathways in statin users having a role in inflammation or immune activation [42]. As such, well-recognised anti-inflammatory effects of statins in atherosclerosis research together with these findings from different study populations (using a variety of methods to assess prostate inflammation) provide suggestive evidence for a potential role for statins in reducing intraprostatic inflammation.

## Limitations of mechanistic insights from observational studies

Although potential mechanisms discussed above are grouped into lipid-mediated and non-lipid-mediated categories, it is important to note that statins have pleiotropic effects, and so these may not be mutually exclusive. Mechanisms are challenging to tease apart in epidemiological studies, and country-specific prescribing patterns and differences in treatment protocols may hinder direct comparison of findings from various studies.

One major consideration is whether it is appropriate to analyse statins together as a class or whether their effects and mechanisms need to be considered according to the statin type or subgroup. While many studies reported the prevalence of use of various statin types and doses (previously summarised in [5]), most were too small to tease apart which type and dose of statin may show greatest benefit. A Canadian registry-based analysis of statin subgroups reported a slightly stronger association of hydrophilic statins with lower prostate cancer-specific mortality (HR 0.68, 95% CI 0.53–0.87), relative to lipophilic statins (HR 0.83, 95% CI 0.70–0.98), but both estimates were statistically compatible with each other [42]. Potentially stronger associations of hydrophilic statins with prostate cancer-specific mortality were supported by a large registry analysis of patients with advanced prostate cancer following ADT that showed somewhat more pronounced associations among users of hydrophilic statins, compared to users of lipophilic statins [43]. However, other large studies reported similar associations across different statin subgroups and types [11, 44, 45]. While future studies should continue to examine associations of statin type and subgroup with prostate cancer, mechanistic insights from laboratory studies may be needed to guide these analyses in epidemiological studies.

In addition to statin type, few observational studies have been able to accurately account for variations in serum cholesterol levels throughout statin therapy. The previously mentioned ARIC study by Mondul et al. performed a stratified analysis by pre-statin serum cholesterol level (normal versus high) and found similar associations between statin use and fatal prostate cancer in both groups [9]. Murtola et al. recorded baseline total cholesterol for patients in a Finnish prostate cancer cohort, similar between statin users and non-users, and reported no effect modification by serum cholesterol of associations between statin use and survival [11]. Whether these findings support the greater importance of non-lipid mediated direct effects of statins, or merely reflect crude measurements of serum cholesterol in these studies is unknown. Serum cholesterol is challenging to measure accurately, varying over time and by fasting status and is often missing in retrospective studies. Future studies tracking prostate cancer patient serum cholesterol at baseline and throughout statin treatment could be beneficial for a better understanding of the influence of cholesterol-mediated statin anti-cancer mechanisms in prostate cancer biology.

## Precision prevention with statins: who might benefit?

Given a cardiovascular risk profile that places most men with prostate cancer at high cardiovascular event risk [46], these men have an on-label indication for guideline-based cardiovascular disease prevention with statins. However, are there certain groups of men who could benefit more than others with respect to prostate cancer-specific outcomes?

As a first step towards answering this question, several studies explored the association between post-diagnosis statin use and prostate cancer-specific mortality, stratified by tumour characteristics at diagnosis. An analysis of the Health Professionals Follow-up Study reported that post-diagnosis statin use was not associated with lower prostate cancer-specific mortality overall (HR 0.97; 95% CI, 0.72–1.31), but found an inverse association among men diagnosed with higher stage prostate cancer (stage T2 and above; HR 0.65; 95% CI 0.43–0.97), not present among men with stage T1 disease (HR 1.26; 95% CI 0.84–1.87) [47]. An observational analysis of men with metastases at diagnosis in the Finnish Randomised Study of Screening for Prostate Cancer found no association between post-diagnosis statin use and prostate cancer death (HR 0.97; 95% CI 0.71–1.32) [11]. A Veterans Administration study found that the inverse association between post-diagnosis statin use and biochemical recurrence among men undergoing surgery for early-stage disease tended to be slightly stronger among men with higher grade cancers (≥4 + 3) (HR 0.51; 95% CI 0.28–0.95) vs. low-grade (≤3 + 4) (HR 0.73, 95% CI 0.52–1.03) [48]. Similarly, a Korean study found that the association between post-diagnosis statin use, and risk of post-surgery biochemical recurrence was stronger among prostate cancer patients with high-grade disease [49]. As such, advanced and aggressive prostate cancer could be considered the most promising clinical setting, but it is unclear which molecular subgroup of men with prostate cancer might benefit.

Examining associations with subgroups defined by pathological and molecular features could highlight not only the most statin sensitive prostate tumours but could also bring to light new molecular targets that may be susceptible to other treatment modalities. Efforts to identify molecular biomarkers of prostate tumours that may be particularly susceptible to statins are now underway, which would enable more precise identification of men predicted to benefit. Within the prospective Health Professionals Follow-up Study, we reported a lower risk of PTEN-null prostate cancer among cancer-free men who used statins compared to non-users (HR 0.40; 95% CI 0.19–0.87) and no association between statin use and PTEN-intact prostate cancer (HR 1.18; 95% CI 0.95–1.48) [8]. This finding is in keeping with experimental evidence supporting an enhanced reliance on cholesterol in tumours with PTEN loss and subsequent upregulation of PI3K signalling, and thus a potentially increased sensitivity to cholesterol-targeting [50]. The association between statin use and prostate cancer did not vary by *TMPRSS2:ERG* fusion (ERG) status [8]. A study of a Finnish radical prostatectomy series suggested that the association between statin use and risk of recurrence could be modified by tumour Ki67 and ERG expression, with a lower risk of recurrence among men with higher Ki67 proliferative index, and without ERG expression, among many potential effect modifiers explored [51]. As ERG status appears to modify the association of several lifestyle factors with prostate cancer risk and survival, such as obesity [52], height [53], physical activity [54], and lycopene intake [55] further research is needed to understand the effect of statin use in this subgroup.

## Current treatment landscape: statins and androgen deprivation therapy

As statins are associated with reduced risk of advanced prostate cancer and androgen deprivation therapy (ADT) is the main treatment for this patient subgroup, it is not surprising that recent investigations have focused on statins in the context of patients managed with ADT (Table 1). A 2017 analysis of the Finnish Randomised Study for Prostate Cancer Screening found that post-diagnosis statin use was more strongly associated with reduced prostate cancer-specific mortality in men undergoing ADT, relative to other treatment modalities [11]. Subsequent studies focused on ADT-treated men only, and overwhelmingly support an inverse association between statin use and prostate cancer-specific mortality in the context of ADT (Table 1).

**Table 1:** Published studies evaluating associations between statin use and prostate cancer outcomes in the setting of ADT, ordered by proportion of primary ADT.

First Author, ref.	Study setting	Study years	*n*	Statin users and type	ADT definition	Exposure	Outcome	Result
Murtola et al. [11]	Finnish Randomised Study of Screening for Prostate Cancer	1996–2012	2649 ADT-treated patients; 617 deaths from prostate cancer over 7.5 years median follow-up	3059 (47%) statin users; simvastatin (78%), atorvastatin (36%)	17% primary ADT; ADT type not specified	Pre-diagnosis statin use (use vs. non-use)	Prostate cancer-specific mortality	HR 0.86; 95% CI 0.69–1.07
						Post-diagnosis statin use (time-dependent variable)	Prostate cancer-specific mortality	HR 0.74; 95% CI 0.59–0.94
Peltomaa et al. [57]	Finnish Randomised Study of Screening for Prostate Cancer	1996–2015	4428 ADT-treated patients; 834 deaths from prostate cancer and 1565 PSA relapses over 6.3 years median follow-up	2544 (48%) statin users during follow-up period	55% primary ADT; LHRH agonists/antagonists (81%), AAs (57%) and/or bilateral orchiectomy (9%)	Pre-ADT initiation statin use (use vs. non-use)	Prostate cancer-specific mortality	HR 1.12; 95% CI 0.96–1.31
							All-cause mortality	HR 1.13; 95% CI 1.02–1.25
						Post-ADT initiation statin use (time-dependent variable)	PSA relapse	HR 0.73; 95% CI 0.65–0.82
							Prostate cancer-specific mortality	HR 0.82; 95% CI 0.69–0.96
							All-cause mortality	HR 0.84; 95% CI 0.76–0.93
Hamilton et al. [61]	Canadian Cancer Trials Group (CCTG) PR-7 trial of intermittent vs. continuous ADT in men with biochemical recurrence after radiotherapy	1999–2005	1364 continous or intermittent ADT-treated patients; 513 deaths, 219 from prostate cancer over 6.9 years median follow-up	287 (42%) statin users in intermittent ADT arm, 298 (44%) statin users in continuous ADT arm	0% primary ADT; Continuous ADT arm: LHRH agonist combined with non-steroidal AA or orchiectomy; Intermittent ADT arm: LHRH agonist combined with non-steroidal AA	Statin use concurrent with ADT (time-dependent variable)	CRPC	HR 0.87; 95% CI 0.71–1.06
							Prostate cancer-specific mortality	HR 0.65; 95% CI 0.48–0.87
							All-cause mortality	HR 0.64; 95% CI 0.53–0.78
Anderson-Carter et al. [63]	National US Veterans Administration database	2000–2016	87,346 ADT-treated patients; 4 752 prostate cancer deaths; median follow-up not reported	53,360 (61%) statin users	Men on short-term ADT (≤6 months) or using ADT in combination with radiation excluded, minimising number of men on primary ADT; LHRH agonists (Leuprolide, Goserelin), non-steroidal AAs (bicalutamide, flutamide and nilutamide)	Statin use concurrent with ADT (minimum 6 months use during study period vs. non-use)	Skeletal-related events (SREs)	HR 0.64; 95% CI 0.59–0.71
							Prostate cancer-specific mortality	HR 0.56; 95% CI 0.53–0.60
							All-cause mortality	HR 0.66; 95% CI 0.63–0.68
Wu et al. [43]	Taiwan Cancer Registry	2008–2014	5749 patients receiving ADT alone in first year after diagnosis; 2 259 deaths, 1495 from prostate cancer over an average of 3.6 years follow-up	2171 (38%) statin users; 40% atorvastatin, 22% rosuvastatin, 12% simvastatin	Majority primary ADT; LHRH antagonists/agonists alone or in combination with AA	Post-diagnosis statin use (defined as anyone prescribed statins for >28 days; modelled as a time-dependant variable)	All-cause mortality	HR 0.75; 95% CI 0.68–0.82
							Prostate cancer-specific mortality	HR 0.77; 95% CI 0.69–0.86
Harshman et al. [64]	US institutional clinical database	1996–2013	926 ADT-treated patients with hormone sensitive prostate cancer; median follow-up of 5.8 years	283 (31%) statin users at ADT initiation	5% primary ADT; ADT type not specified	Statin use vs. non-use at ADT initiation	PSA relapse	HR 0.83; 95% CI 0.69–0.99
Mikkelsen et al. [62]	Two Danish Urological Departments	2007–2013	537 advanced or metastatic prostate cancer patients, primarily treated with ADT alone; 315 prostate cancer deaths over 5.7 years median follow-up	141 (26%) statin users at time of diagnosis	100% primary ADT; bilateral orchiectomy or LHRH agonists/antagonists	Statin use vs. non-use at diagnosis	Prostate cancer progression (CRPC or prostate cancer-specific mortality)	HR 0.98; 95% CI 0.72–1.32
							All-cause mortality	HR 1.11; 95% CI 0.82–1.50

By inhibiting androgen synthesis in the testes, ADTs lower systemic androgen levels, limiting androgen signalling pathways driving prostate cancer survival and proliferation. However, tumour progression inevitably occurs in spite of low availability of circulating androgens through a variety of documented mechanisms including intratumoral androgen synthesis from cholesterol [56]. As such, it has been hypothesised that statins may synergise with ADT by blocking accumulation of intratumoral cholesterol thereby reducing the substrate for de novo androgen synthesis within the prostate [57]. Given that more advanced tumours upregulate enzymes necessary for de novo androgen synthesis [58–60], it may be that statins have a stronger effect in later-stage tumours, enriched in the context of salvage ADT. Indeed, inverse associations reported by the various observational studies completed to date (Table 1) appear slightly stronger among patients receiving salvage ADT, typically administrated later during the disease course compared to primary ADT. Among studies where the majority were using salvage ADT, estimates for statin use in association with prostate cancer-specific mortality ranged from HR 0.65 (95% CI 0.48–0.97) [61] to HR 0.86 (95% CI 0.69–1.07) [11], whereas among studies in the context of majority primary ADT, estimates ranged from HR 0.82 (95% CI 0.69–0.96) [57] to HR 0.98 (95% CI 0.72–1.32) [62]. These estimates could support a biological mechanism or be the result of greater difficulty with controlling time-varying confounding in settings of salvage ADT, where data on patient and tumour characteristics are typically known only at diagnosis.

With a view to understanding which point in clinical progression that the tumour may be most sensitive to statins, a number of studies reporting lower prostate cancer-specific mortality in statin users (Table 1) performed secondary analyses stratified by tumour features. Peltomaa and colleagues found no difference in the association between post-ADT statin use and prostate cancer-specific mortality when stratified by tumour risk group [57]. Anderson-Carter et al. reported similar associations between statin use and outcomes when they restricted to men with higher PSA (>10 ng/ml) relative to the entire cohort [63]. An analysis of the Taiwan Cancer Registry found similar results among with men T3/T4 disease and those with metastatic prostate cancer at diagnosis, relative to the full cohort [43]. Finally, an analysis of clinical datasets found that effect estimates for the association between statins and PSA relapse were similar for those starting ADT with biochemical recurrence alone vs. those with evidence of metastatic disease [64]. As such, there does not currently appear to be strong observational evidence supporting a stronger benefit of statins for specific tumour risk groups within the context of ADT.

Aside from blocking intratumour cholesterol ester accumulation, which may primarily affect advanced tumours with the necessary enzymatic machinery to synthesise intratumoral androgens, an alternative hypothesis as to why statins may synergise with ADT lies in their potential effect on adrenal androgens such as dehydroepiandrosterone (DHEAS), a precursor to the more potent dihydroxytestosterone (DHT) and testosterone. As adrenal androgens continue to be synthesised despite orchiectomy or pharmaceuticals that modulate gonadotropin-releasing hormone, which largely target testicular androgen synthesis, they are thought to be one of the mechanisms of tumour resistance to ADT [65]. A pre-specified post-hoc analysis of the Finnish clinical trial of atorvastatin in prostate cancer found downregulation of adrenal androgens in both serum and prostate tissue of men randomised to atorvastatin treatment [66] supporting the ability of statins to reduce a source of androgens mostly untargeted by current therapies. In addition to reduction of adrenal androgen synthesis, statins may compete with DHEAS for tumour uptake via the SLCO transporters [64]. As such, these could potentially be mechanisms whereby statins complement ADT therapy for less advanced prostate cancers. Moreover, this may explain why statins concurrent with ADT have been demonstrated to have a stronger benefit than statin use prior to ADT. As the current focus for most studies to date has been the ability of statins to prolong time to castration-resistant prostate cancer (CRPC), more information is needed to determine the optimal time for statin use in patients receiving ADT; whether it is beneficial to start statins prior to or during ADT and if, and how long, statins should be continued when ADT ceases or palliative care is commenced. A recent study within the Finnish Randomised Study of Screening for Prostate Cancer examined timing of statin use in relation to ADT, reporting an inverse association between statin use and prostate cancer-specific mortality when statin exposure was defined during ADT (HR 0.82; 95% CI 0.69–0.96), but not when statin use was defined before starting ADT (HR 1.12; 95% CI 0.96–1.31) or in the first year following ADT initiation (HR 1.02; 95% CI 0.85–1.24) [57, 67]. Additionally, more targeted investigations are needed to evaluate potential benefits of statins alongside ADT specifically in CRPC patients as there are currently limited or inconsistent studies to adequately examine the potential for this combination in prolonging CRPC patient survival over ADT alone.

Second generation anti-androgens, including abiraterone acetate, interfere with the androgen signalling axis in multiple organs including the adrenal glands as well as the prostate itself. As such, the aforementioned downregulation of adrenal androgens by statins suggests a mechanism whereby statins could complement downregulation of adrenal androgens by abiraterone to improve prostate cancer outcomes. As statins compete with abiraterone for SLCO-mediated influx there were initial concerns that statins may be antagonistic to abiraterone and therefore interfere with treatment efficacy. However, a US hospital-based cohort study found a trend towards longer duration of abiraterone response among men with CRPC using statins (HR 0.79; 95% CI 0.57–1.09) [68]. The retrospective, hospital-based cohort study STABEN evaluated the association of statin use in mCRPC patients already receiving abiraterone or enzalutamide [69] and reported pronounced inverse associations between statin use and overall mortality risk (HR 0.47; 95% CI 0.35–0.63) and more frequent PSA declines (>30%) within the first 3 months (OR 1.63; 95% CI 1.03–2.60). Another retrospective hospital-based study of 187 patients receiving anti-androgens for mCRPC found statins were associated with longer overall survival (HR 0.40; 95% CI 0.27–0.59) [69]. Further investigations are required to study different durations of statin use prior to anti-androgen initiation at advanced and castrate-resistant stages of the disease to ascertain the relevant window for statin treatment initiation if it was to be used in this context.

### Future directions

To date, the observational data summarised in our review shows a narrowing of focus for the promise of statin for prevention of all prostate cancer to prevention of fatal prostate cancer. Results showing an inverse association between statins and fatal prostate cancer risk are promising. While this is particularly encouraging given these patients currently have limited effective treatments and shorter survival times, biomarkers may help identify and subsequently target the molecular subgroups that are most likely to benefit from statin therapy. Given that statins are comparatively safe, in addition to support for improved prostate cancer-related outcomes in statin users, it could be argued that most men with prostate cancer should receive a statin. A central consideration here is that men with prostate cancer, particularly those receiving ADT [70], are at simultaneous risk for adverse cardiovascular and prostate cancer outcomes. However, it is vital to identify the patients for which statin therapy would have greatest impact as perhaps not all men will benefit from the same dose, duration, or type of statin, and some or many may not benefit at all.

It is critical that these questions be evaluated using randomised-controlled trials. With advances in molecular profiling of prostate cancer, resulting biomarkers may aid in more efficient selection of participants for clinical trials of statins as well as become appropriate intermediate endpoints in such trials.

## Conclusions

As one of the world’s most prescribed medications, statins are well-established and clinically safe drugs. For prevention and treatment of prostate cancer, as summarised in this review, the most pronounced and robust associations have been observed between statin use and outcomes related to advanced prostate cancer. A key consideration is the high burden of cardiovascular disease among men with prostate cancer [46, 71], with modestly higher cardiovascular disease risks among men typically treated with ADT [70]. Thus, even without definitive data on statin effects on prostate cancer, the overall risk-benefit balance already favours initiation and continuation of statins among many men simply due to on-label indications and clinical guidelines for prevention and treatment of cardiovascular disease.

Our review shows that statins are not a cure-all, but the hype surrounding this class of drugs is understandable. Prostate cancer research motivated by potential statin effects has advanced our understanding of prostate cancer biology with regards to both cholesterol and lipid metabolism (see partner review of mechanistic data) [2] as well as between-tumour heterogeneity, with potential implications about which subsets of men and tumours might benefit. Evidence supports potential benefits of statin use alongside several of key therapies, such as ADT, that are employed against advanced prostate cancers.

With many observational studies supporting the hypothesis that statins may protect against fatal prostate cancer, there is hope that statins could form part of a multipronged therapeutic strategy. Ultimately, the focus should be on testing this hypothesis in patients using randomised-controlled trials. Furthermore, translational research integrating epidemiological data and lab-based investigations could help identify biomarkers of statin sensitivity. Such biomarkers would be beneficial to define molecular subgroups for subsequent examination in observational and trial-based settings.
